# Valued experiences of graduate students in their role as educators in undergraduate training in Ugandan medical schools

**DOI:** 10.1186/s12909-017-1073-2

**Published:** 2017-11-25

**Authors:** Godfrey Zari Rukundo, Jannat Kasozi, Aluonzi Burani, Wycliff Byona, Claude Kirimuhuzya, Sarah Kiguli

**Affiliations:** 1 Department of Psychiatry, Mbarara University of Science and Technology, Mbarara, Uganda; 2 Department of Nursing, Mbarara University of Science and Technology, Mbarara, Uganda; 3 Kampala International University, Western Campus, Mbarara, Uganda; 4 Wipa Research Consults, Kampala, Uganda; 5 Department of Paediatrics and Child Health, Makerere College of Health Sciences, Kampala, Uganda

**Keywords:** Graduate students, Undergraduate training, Medical schools, Uganda

## Abstract

**Background:**

In most medical schools, graduate students, sometimes referred to as graduate teaching assistants, often participate in the training of undergraduate students. In developing countries like Uganda, are typically involved in undergraduate training. However, prior to this study there were no standard guidelines for this involvement. At the same time, the views and experiences of the graduate students in their role as educators had not been documented. Therefore, the aim of this study was to explore the views and experiences of graduate students about their involvement in undergraduate training in three Ugandan medical schools. The findings of this study will contribute to the development of policies for training in Ugandan medical schools.

**Methods:**

This was a qualitative study in which thirty in-depth-interviews were conducted among second and third year graduate students in three Ugandan medical schools in the MESAU consortium (Medical Education Services to all Ugandans) including Mbarara University of Science and Technology, Makerere College of Health Sciences and Kampala International University, Western Campus.

**Results:**

All graduate students from all the three medical schools viewed their involvement in undergraduate training as important. The study also revealed that graduate students increase available human resources and often compensate for the teaching missed when senior educators were absent. The graduate students expressed important views that need to be considered in the design of educational programs where they are to be involved. The respondents also reported a number of challenges in this undertaking that included lack of motivation, lack of orientation and having heavy workloads. The presence and commitment of senior educators to guide and support the graduate students in teaching activities was viewed as one significant intervention that would increase the effectiveness of their educational contributions.

**Conclusions:**

Graduate students enjoy their involvement in the training of undergraduate students despite the various challenges they face. In some departments, the involvement of postgraduate trainees is critical to the viability of undergraduate medical training.

**Electronic supplementary material:**

The online version of this article (10.1186/s12909-017-1073-2) contains supplementary material, which is available to authorized users.

## Background

Graduate students, sometimes referred to as Graduate Teaching Assistants (GTAs), are typically involved in undergraduate training, especially in circumstances where there are limited human resources [1, 2]. This educational involvement can provide opportunities for career development in medical education as well as enhance confidence and responsibilities as medical leaders especially in low and middle income countries [3, 4].

Despite the benefits, the task may be challenging to GTAs due to time lost that could otherwise be spent in research or personal studies [5]. Although this educational involvement is common, there are no available standard guidelines or best practices for GTAs providing undergraduate medical education in low and middle income settings [6]. Previous research has largely been conducted in high income and developed countries [2, 7–13] with no available literature on the experience of GTAs in developing countries. The relative dearth of literature in this regard may reflect a lack of prioritization of medical education research in developing countries. This paper describes views and experiences of GTAs from selected Ugandan medical schools about their role in undergraduate training in Ugandan medical schools. The findings of this study will contribute to the development of policies and guidelines for graduate medical education.

## Methods

### Study design

This study explored the views and experiences of graduate students in their role as GTAs in three Ugandan medical schools. Using qualitative methodology, in-depth interviews were conducted among second and third year medical graduate students at three Ugandan medical schools selected from the members of the consortium for Medical Education Services to All Ugandans (MESAU). MESAU members consist of Mbarara University of Science and Technology, Makerere University College of Health Sciences, and Kampala International University - Western Campus. Two university members of the consortium (Gulu and Busitema Universities) were excluded from the study because they did not have graduate medical programs at the time of the study.

### Study participants

The participants were a mixture of 30 male and female GTAs pursuing various clinical and biomedical graduate programmes offered in the three medical schools. Majority of the participants were privately sponsored and were not part of university staff capacity development. GTAS were primarily from Uganda, although some were from other African countries. All of the GTAs were from low-income countries.

### Data collection

A total of thirty in-depth interviews (IDIs) were conducted in three Ugandan medical schools, with 10 interviews per school Additional file 1. The IDIs were captured using a voice recorder. Hand written notes were also taken during the IDIs by two separate researchers to capture meeting proceedings as well as specific details, such as body language of the respondents. The recorded IDIs were later transcribed and reconciled with the hand-written notes. Saturation of themes was reached after 10 GTAs were interviewed from the 3 universities such that no new ideas were expressed. No new interviews were conducted after this. The views and experiences of the interviewed graduate students are the focus of this paper.

### Data analysis

Methodological principles in qualitative studies as outlined by Sargeant et al. and Hanson et al. informed the data analysis [11, 12]. Themes were indentified reviewing each transcript in detail. Broad themes were identified according to sites and within pooled data. Data was organized into concepts and tentative explanations were generated. Exploration of the relationships between concepts allowed for the emergence of thematic trends and conclusions from the data.

## Results

After data analysis, six themes emerged as follows:

### Graduate students’ views on their involvement in undergraduate medical education

Involvement of GTAs was perceived by all the graduate students as beneficial experience. They felt that it was like “paying back” given their own experience as undergraduate medical learners. Power differentials were not identified in that GTAs did not report being intimidated by their seniors. The GTAs reported that they were more available than the senior staff to attend to the undergraduate students.

*‘Our teachers have a big load and if we are to learn from the same teachers, we must be part of the people to offload them’ (MakCHS student)*

The graduate students regarded their participation in undergraduate medical training as an opportunity to practice what they have studied and acquire more teaching skills. The GTAs saw the teaching opportunity as a way of consolidating knowledge and also indicated that they did not want to appear unprepared. In part, the fear of embarrassment motivated their learning. Furthermore, graduate students also regarded it as one way helping them to build their own confidence as future teachers since teaching would help them to bridge knowledge gaps.

*“… a hand that washes, also becomes clean” (MUST student)*

*“....When you teach others, you understand what you learn better” (MakCHS student).*

### Contributions of GTAs to undergraduate training in Ugandan medical schools

GTAs reported that in their capacity, they make several contributions to undergraduate health professional training in the current Ugandan situation. For example, they form part of the team of tutors/teachers involved in conducting tutorials and provide details in practical sessions that the senior staff have no time to teach. In addition, graduate students participate in overview of lectures, setting and marking of examinations and acting as role models for the graduate students.

*“… We help them not to fall in to the ditches we fell in; so we guide them and explain in lay man’s language” (MakCHS student)*

### Preparation of graduate students to take on the role of teaching undergraduate students

Although the graduate students are involved in undergraduate training, the majority of the graduate students in all the institutions reported no formal preparation for this role. GTAs reported relying on their personal experiences to approach the educational tasks given to them.

*“… Involvement in undergraduate teaching is like one of the unwritten aspects of the contract for graduate students.” .....“ We do not get any formal training. Yet, ‘you might be a good doctor but not necessarily a good teacher” (MUST student)*

*“… you just find yourself when the undergraduates are waiting for you to attend to them; they may be eager to learn but you find that no one else is attending to them” (MakCHS student)*

Some departments in the medical schools provided orientation and support to the graduate students before involving them in undergraduate training. For example, in some departments graduate students were given the teaching role after spending one first semester in the Masters’ program. In other departments, senior staff would review materials prepared by the graduate students prior to teaching sessions. A few departments provided some formal training to graduate students on how to teach.

*“… we have done a few courses on presentation skills, eloquence and how to teach” (MakCHS student)*

### Attitudes of senior educators (specialists) towards graduate students in their role as educators

The GTAs had divergent views concerning the attitudes of senior educators towards graduate students in their role as educators in undergraduate training. Some had negative impressions while others had positive experiences.

*“… Well I think they view us as helping hands; … they know it is their role and not ours to teach undergraduate students”* (MakCHS student*).*

“ *… My professor sees it as my duty to learn because at the end of it all once I graduate, I have to be able to transfer knowledge to other people” (MUST student)*

*“… In a way it appears like we are ‘tools’. If a lecturer has a class and it is somehow an inconvenient time or he is caught up by time, they know there is always a graduate student who can take the students through that topic, so in a way sometimes we appear like mercenaries or some easy labour somewhere that they can fall back to” (MakCHS student)*

### How the graduate students are affected by their involvement in undergraduate training

Under this theme, a number of issues and challenges emerged. One of the main challenges reported by the graduate students in their role as educators was conflict of time and commitment as reflected in the quotes below:

*“…Time is not enough. Programs collide and there is conflict of priorities” (KIU student)*

*“… As a graduate student, there are a lot of things to do. You always want to use your time in graduate work. Most of the time we are inconvenienced like during exams; they want us to also be on the team that is examining and marking tests. They can also call us to lecture students when we are having our own lectures to attend” (MakCHS student)*

Additionally, GTAs clearly stated that time spent in teaching detracted from their postgraduate requirements. Teaching responsibilities were not viewed as part of the graduate curriculum and therefore were not able to be accounted for. The quotation below reveals this sentiment:

*“… You really find that you would have used your five hours after lunch or before lunch for your private reading to understand other things or research, but then you find yourself having to be with them for those hours. So it consumes much of our time because of the poor preparation” (MakCHS student)*

Lack of monetary benefit to graduate students was another major challenge as is portrayed in the following quotes:

*“… There is no motivation behind it, yet it needs putting in an extra time, say coming in early and leaving late and required to read different things” (KIU student)*

*“… We also feel like we are doing someone else’s work. ……that is why most graduate students miss delivering of the lecture saying somebody is paid to do this work and not me. Logistically, it is not friendly at all. No monetary motivation” (MakCHS student)*

On the other hand, the GTAs reported personal gains being involved in undergraduate training. Some of them reported improved confidence, communication skills, and aided in the acquisition of current knowledge. One participant stated the following:

*“… It has kind of built my confidence in delivering these principles. So that confidence helps me understand these things more and also look out for more details…. You have to be equipped with most recent information before going to those classes. And it also helped me to develop my communication skills…” (MUST student)*

Furthermore, the respondents reported that involvement in undergraduate training enriched their Curriculum Vitae as it pertained to having experience as medical educators. Participants also stated that their experience prepared them to be future educators.

*“… It prepares us to learn how to speak to people; we learn how to pass on skills. It prepares us to learn how to deal with students, because all of them have a number of issues and by the time we are through with them of course we may know a student who is ‘slippery’ (unreliable) and one who is not and how to help each of them” (MUST student).*

*“…There are things we did not take seriously when we were studying. But, now being the resource persons, we have to prepare and have to read more” (KIU student)*

Involvement in undergraduate training helped the GTAs to develop leadership and organization skills. Participants stated the following:

*“… As a teacher, you are a role model, so it does really prepare you in such a way that as you are doing it, you are being a leader first of all. So leadership skill and organization, all those skills are being developed in you. And also as I have said, as a doctor you need to pass on those skills to the undergraduates. So it is nurtured in you” (MakCHS student)*

*“… It gives you the leadership part of it. You become a leader automatically. Passing on knowledge is really something that is superior to just being a clinician. You feel you are contributing to some one’s life” (MUST student)*

The GTAs revealed that they learned important interpersonal skills through teaching undergraduates. The following quotes highlight this.

*“… you learn what I would call interpersonal skills, because you go to a class and it has so many people, you need to know how you are going to control that class, how you are going to keep them attentive, you need to know how to control the noise, the murmuring, you need to know at what point you are going to switch to another thing and how to prepare your presentation” (MakCHS student)*

Respondents stated that they learned how to appropriately respond to questions asked by undergraduate students.

*“… When you look at it, sometimes you can think that teaching is easy but it’s not. So sometimes you can teach and no one understands but the way they are training is you teach............ okay the way you pass on the information, how can you asses that someone has understood, how do you make sure that everyone is paying attention like.......... I think that really prepares you to be a teacher” (MakCHS student)*

The respondents reported that involvement in undergraduate training widens their perspective of the future.

*“… It opens up your spectrum. Because when I came, I thought all I wanted to be was be a gynecologist and get out of here but you find that this teaching helps you in one way or the other. It shows you the academic part of it. It gives you other options, you cannot only be a pediatrician, gynecologist, but you can be a lecturer or even can contribute in the field of research. So I think to me it opened up my spectrum” (MUST student)*

### GTAs identify changes to improve their involvement in undergraduate medical education

The respondents identified how their involvement could be improved. They suggested that senior educators were often not as visible or available during undergraduate teaching sessions. The GTAs indicated a desire for senior staff to be present and to engage in the teaching activities they are being remunerated for. This is illustrated in the following quote:

*“… You see what I think in my opinion, it should be an individual part and effort, if you are a lecturer and you are supposed to teach at a particular time, they should be involved, if you are paid to be a lecturer, you should always be around, not being in the private clinic seeing other patients so it is really important because this also affects post graduate students” (MakCHS student)*

GTAs also described situations where they felt unable to meet the educational needs of the many undergraduate medical students. They suggested working with a smaller number of learners so as to better manage the educational demands. Currently, this is not the practice. A GTA is expected to attend to whoever requires assistance.

*“... if for graduate students are required to teach undergraduates, then they need to assign us students and they should not be more than 3 students because the more the number of students to attend to, the less benefit they get” (MakCHS student)*

GTAs also reported difficulties with workload as it pertained to teaching responsibilities.

*“… The graduate students’ workload should not be like for the other staff on the payroll. They should have less to teach and more time to concentrate on their development” (KIU student).*

*“… I think the undergraduate students should be given particular hours for contact with us, not all the time” (MUST student)*

Furthermore, GTAs recommended that involvement of graduate students in the training of undergraduate students should be well organized and streamlined.

*“… the system should design a program on how to handle teaching of undergraduate students by post graduate students; it should be well organized” (MakCHS student).*

Another suggestion was that graduate students require more supervision from the senior staff. It was clear from the multiple responses that this type of support is commonly missing in most departments.

*“… The seniors should be a little bit more supportive. You find that all the tutorials are scheduled for specialists but they do not come to conduct the tutorials. Graduate students end up doing the work alone” (MUST student)*

*“… it should not to be seen as an obligation, because really if a specialist is paid to do that, he should do it; but if he finds out that a graduate student has not done it, that becomes a problem” (MUST student).*

The GTAs also recommended they should receive remuneration for their efforts.

*“… Surely you come and you teach, ‘you get this kind of view like you are doing some one’s work and you are not being paid’. But if there could be motivation like being paid some money to do research, it would be better. Most times the graduate students feel cheated”. (MakCHS student)*

Although the GTAs recognized that money would be an incentive, they agreed other factors motivated them, including:

*“…. The motivation does not necessarily need to be money; it could be other rewards that can be developed for that. The point is really that there is something you get out of it. But for those who do not see that clearly, there is something, may be they have a course they are doing, most people like certificates that is ok. Well as those who need the money, let them be given the money. They need to figure out in each department what works best, what translates into remuneration for a person” (MakCHS student)*

The GTAs suggested that senior staff should be paid well, making it more likely that they would be available to teach undergraduate students.

*“.. May be they should pay more money to these senior lecturers so that they become motivated, it is a reality, these things are really happening” (MakCHS student)*

The respondents further argued that there was a need for graduate students to be given orientation to how institutional systems affected teaching responsibilities. They also agreed that having access to guidelines prior to the start of teaching would be helpful.

“… *I think they need to give us an outline of the procedures one should go through while teaching the students because really you learn on course or duty so you really don’t know how the student should benefit from what I am going to talk or teach, so they need to give at least an outline on how they are supposed to do the teaching before you can begin the actual teaching for example someone comes and gives you an outline saying ‘you know when you are teaching these students, you have a lot of knowledge, you need to begin with your introduction” (MakCHS student).*

Some GTAs suggested that the use of postgraduate trainees as primary educators for medical students “cheated” undergraduates from receiving an optimal education. Their view was that undergraduate students deserve the quality teaching they have paid for, which includes education from senior lecturers.

“… *I think these students are clients of the university. They come here for specific services. Some of them are paying for themselves and they come here to get value for their money. So you want to get the best services from the right person. Sometimes graduate student is not the best person for the subject you want to learn” (MakCHS student)*

GTAs suggested that receiving instruction on teaching and communication skills before being asked to teach undergraduates would be beneficial.

*“… Teaching is not going there and giving knowledge, you need to know what you are going to teach but we tend to learn on the job. As time goes on, you feel you are better, even the students will start saying that so and so is better now that we are in the fifth year so I think that should be done” (MUST student)*

*“… To improve, I think the university should develop a culture of orientation. Teaching is like a profession, not everyone can pass on the knowledge even if you have it. They should orient the postgraduate students on the expectations that are required for one to teach” (MUST student)*

Finally, the GTAs suggested having access to teaching materials and teaching facilities in order for their experience to be both enjoyable and effective.

*“… We need teaching facilities like internet, equipment and exchange programmes” (KIU student)*

## Discussion

This study explored the views and experiences of GTAs concerning their involvement in undergraduate medical education in three Ugandan medical schools. All GTAs viewed their involvement in the teaching and learning of undergraduate students as a valuable contribution. Their involvement was perceived to be appreciated by the undergraduate students. GTAs also believed that they played an important mentoring role. This relationship was described as facilitating learning [6].

Although the GTAs are interested in undergraduate training, they need to be supported by senior educators in order to be able to do a better job. Suggestions included training, mentorship and regular supervision. The role of GTAs serves to increase available human resources in the medical schools, which are largely understaffed in a majority of the departments [6, 14]. Despite the described benefits, this involvement is associated with challenges for both GTAs and undergraduate students within the same medical schools. Considering possible solutions for the encountered challenges is critical.

Previous studies have reported similar findings in other settings [15]. One of the key facilitators of the positive outcomes is the effective communication between GTAs and undergraduate students. In low-income countries, undergraduate and postgraduate learners are usually not far apart in age which allows for a lateral, peer based relationship as compared to relationships with senior educators [6]. Effective communication facilitates learning, supervision and mentorship [6]. In the process of mentoring and supervising undergraduate students, the GTAs reinforced their own learning. Additionally, they developed confidence and skills that can be beneficial for future work in academia [15]. The mentorship relationship between the graduate and undergraduate students is essential for development of deeper learning in different subjects. This same kind of relationship exists when junior teachers mentor each other [16–18]. There are no power differentials and the relationship is mutually beneficial.

Because of the effective involvement of graduate students in teaching and mentoring, the senior staff seem to relinquish teaching responsibilities. Although this is not preferable or desirable, it is a challenge for GTAs to negotiate given the inherent power differential. Although learning is taking place, the GTAs feel they are not experts in the areas they are teaching. GTAs feel as though they need guidance from the senior educators, if they are to do a better job. In the process of carrying out their duties, graduate students may encounter difficulties or make errors. Hence, there is need for senior educators to be present and to support professional development of GTAs [14].

Whereas the involvement of GTAs in undergraduate studies is appreciated, GTAs also have their own educational needs that must be met. For example, GTAs also require sufficient contact with their own educators. Therefore, there should be an organized plan that incorporates attention to GTAs’ own teaching and learning. Development of mentorship programs as well as opportunities to enhance their own pedagogical skills would be helpful [6]. Mentorship or teaching is not specifically addressed in many low-income postgraduate medical training programs, and there is need for training in preparation for these roles [19, 20].

Motivating GTAs in their role as trainers is to give feedback on their performance is also highly recommended as a strategy to improve pedagogical performance. It is recommended that these strategies are engaged in by both GTAs and senior educators. Previous studies have reported that, when appropriate skills are given to GTAs, it motivates them to remain in the training institutions [14]. In a low-income setting such as Uganda, retention may be a serious challenge given the limited resources.

## Conclusions

Graduate students enjoy their involvement in the training of undergraduate students despite the various challenges they face. In some departments, the involvement of postgraduate trainees is critical to the viability of undergraduate medical.

## Additional file

Additional file 1: IDI questions for graduate students. (DOCX 16 kb)

## Abbreviations

GTA: Graduate Teaching Assistant
KIU: Kampala International University
MakCHS: Makerere University College of Health Sciences
MUST: Mbarara University of Science and Technology
