# Supplementary material for: Valued experiences of graduate students in their role as educators in undergraduate training in Ugandan medical schools
Source: BMC Med Educ. 2017 Nov 25;17:231. doi: 10.1186/s12909-017-1073-2 (PMC5702216; doi:10.1186/s12909-017-1073-2)
Supplement: Additional file 1: — IDI questions for graduate students. (DOCX 16 kb) [file 12909_2017_1073_MOESM1_ESM.docx]

**IDI QUESTIONS FOR GRADUATE STUDENTS**

Qn1: What is your view on the involvement of graduate students in teaching of undergraduate students?

Qn2: What contributions do the graduate students make to undergraduate training?

Qn3: In what way were you prepared by the university to take on the role of teaching these undergraduate students?

QN4: In your opinion, how do the seniors (specialists) view the graduate students in their role as teachers?

Qn5: How does your involvement in undergraduate training affect you?

Qn6: In which way does this teaching role prepare you to be a future educator?

Qn7: Have benefited from your involvement in under graduate student teaching? If yes how, if no why do you think you have not benefited?

Qn8: what do you think can be done differently about your involvement in undergraduate training?

Qn9: Any other comments?
